# Comparison of Different Physical Activity Measures in a Cardiac Rehabilitation Program: A Prospective Study

**DOI:** 10.3390/s22041639

**Published:** 2022-02-19

**Authors:** Muaddi Alharbi, Adrian Bauman, Mohammed Alabdulaali, Lis Neubeck, Sidney Smith, Sharon Naismith, Yun-Hee Jeon, Geoffrey Tofler, Atef Surour, Robyn Gallagher

**Affiliations:** 1 The Studies and Consulting Office at the Assistant Minister of Health, Riyadh 11176, Saudi Arabia; 2 Charles Perkins Centre, Faculty of Medicine and Health, University of Sydney, Sydney 2006, Australia; adrian.bauman@sydney.edu.au (A.B.); sharon.naismith@sydney.edu.au (S.N.); yun-hee.jeon@sydney.edu.au (Y.-H.J.); robyn.gallagher@sydney.edu.au (R.G.); 3 Department of the Assistant Minister, Ministry of Health, Riyadh 11176, Saudi Arabia; mal-abdul-aali@moh.gov.sa; 4 School of Health and Social Care, Edinburgh Napier University, Edinburgh EH14 1DJ, UK; lis.neubeck@sydney.edu.au; 5 Division of Cardiology, University of North Carolina, Chapel Hill, NC 27514, USA; scs@med.unc.edu; 6 Royal North Shore Hospital, Sydney 2065, Australia; geoffrey.tofler@health.nsw.gov.au; 7 The National Association for Health Awareness (Hayatona), Riyadh 12466, Saudi Arabia; atef_surour@yahoo.com

**Keywords:** coronary heart disease, physical activity, Fitbit, 6MWT, PASE, responsiveness, interchangeability, cardiac rehabilitation

## Abstract

Concordant assessments of physical activity (PA) and related measures in cardiac rehabilitation (CR) is essential for exercise prescription. This study compared exercise measurement from an in-person walk test; wearable activity tracker; and self-report at CR entry, completion (8-weeks) and follow-up (16-weeks). Forty patients beginning CR completed the Six-Minute Walk Test (6MWT), Physical Activity Scale for the Elderly (PASE), and wore Fitbit-Flex for four consecutive days including two weekend days. The sample mean age was 66 years; 67% were male. Increased exercise capacity at CR completion and follow-up was detected by a 6MWT change in mean distance (39 m and 42 m; *p* = 0.01, respectively). Increased PA participation at CR completion was detected by Fitbit-Flex mean change in step counts (1794; *p* = 0.01). Relative changes for Fitbit-Flex step counts and a 6MWT were consistent with previous research, demonstrating Fitbit-Flex’s potential as an outcome measure. With four days of data, Fitbit-Flex had acceptable ICC values in measuring step counts and MVPA minutes. Fitbit-Flex steps and 6MWT meters are more responsive to changes in PA patterns following exposure to a cardiac rehabilitation program than Fitbit-Flex or PASE-estimated moderate–vigorous PA (MVPA) minutes. Fitbit-Flex step counts provide a useful additional measure for assessing PA outside of the CR setting and accounts for day-to-day variations. Two weekend days and two weekdays are needed for Fitbit-Flex to estimate PA levels more precisely.

## 1. Introduction

Coronary heart disease (CHD) is a leading cause of death [1] and lack of physical activity (PA) has been identified as a substantial contributor to cardiac mortality [2,3]. Evidence-based cardiac rehabilitation (CR) programs that target lifestyle modification to improve PA reduce all-cause and cardiac mortality [4]. Engagement in PA-based CR fosters recovery from a cardiac event and potentially reduces recurrence by improving blood pressure, lipoprotein profiles, and blood glucose metabolism, reducing body weight and depression severity and enhancing psychological health [5,6]. Due to the multiple beneficial effects of PA, CR programs aim to assist cardiac patients to engage in PA for life. The programs also emphasize the need to achieve the recommended amount of PA required for the secondary prevention of cardiac diseases (i.e., PA most days of the week, at least at a moderate-intensity level for 30 min [7] or walking for at least 7000 steps [8]).

Measurement of PA is an essential component of CR programs for determining entry-level functional capacity and PA levels to enable appropriate exercise prescription, and for the evaluation of training and long-term outcomes for CR programs. The conventional in-person Six-Minute Walk Test (6MWT) is commonly recommended to assess exercise capacity, and self-reported surveys or questionnaires are frequently used to assess PA duration and intensity [9]. Although the 6MWT can be affected by several factors (including age, gender, height, and weight), changes in walk distance after clinical interventions are considered as closely representing the true change in patients’ exercise capacity [10]. Furthermore, performance on the 6MWT enables more individualized exercise prescription. However, CR is increasingly being delivered using telehealth and virtual community-based modes that preclude the use of the 6MWT. On the other hand, indirect assessment through self-reported PA measures have the unique advantage of recording PA type at low cost and with ease of administration [11]. However, relying on self-reported PA has a number of limitations. Two systematic reviews demonstrate that most self-reported PA measures are more applicable to epidemiological studies rather than clinical CR settings [12] and overestimate PA levels by around 50% when compared to objective measures [11]. Despite common usage of the conventional in-person 6MWT and indirect PA measures through tracking and self-report questionnaire in CR settings, there is an ongoing need to understand the capacity of these measures to detect meaningful changes in PA over time (responsiveness), and to appropriately support different models of CR delivery, including telehealth and virtual support. Moreover, the current measurement of PA in CR does not accurately address the patient’s overall pattern of engagement in PA [13]. Cardiac patients’ PA behaviors vary over time, with evidence demonstrating that they reached adequate PA levels on rehabilitation program days, but failed to achieve target levels on non-rehabilitation program days [14].

Modern self-managed measures, including wearable PA trackers such as Fitbit-Flex, offer a novel and real-time approach for objectively measuring the overall PA levels of CR participants [15]. In addition, they have the potential to provide feedback to encourage PA and to promote interaction between patients and clinicians through detailed PA tracking, which may lead to better outcomes [15,16]. Further, as revealed through the COVID-19 pandemic, there is a need for telehealth methods that do not require an in-person assessment. Notwithstanding these benefits, the usefulness of wearable activity trackers such as Fitbit-Flex as a measure of PA in response to CR participation has not been well examined.

Our validation study [15] of 48 cardiac patients and family members participating in a CR program compared Fitbit-Flex against an Actigraph accelerometer for measuring step counts and moderate–vigorous physical activity (MVPA). Fitbit-Flex demonstrated high accuracy, sensitivity, and specificity for classifying participants according to whether they met the PA guidelines for step counts and minutes of MVPA. We concluded that Fitbit-Flex has acceptable validity for measuring PA in CR participants.

However, several important questions remain in relation to the measurement of PA using wearable activity trackers such as Fitbit-Flex. For instance, the reliability of Fitbit-Flex for CR patients and its assessment of change during and following CR interventions needs to be established. Evidence of Actigraph reliability demonstrated that in a CR population, two to four days of accelerometer data collection are sufficient to characterize a weekly pattern of PA behavior [17], but additional days of data collection will further increase reliability [18,19]. Including weekdays and weekends in the monitoring period, and adjusting for seasonal effects also improves the reliability of the PA measure [20]. Given the difference in PA behavior patterns, the reliability of Fitbit-Flex to characterize a weekly pattern of PA behavior for CR patients should be investigated. The primary aim of this study is to determine and to compare the measurement and responsiveness of Fitbit-Flex assessed step counts and estimates of MVPA minutes, the self-reported Physical Activity Scale for the Elderly (PASE), and the 6MWT in detecting changes in PA for CHD patients attending CR phase II programs through assessments at the entry of CR, completion of CR and CR follow-up. This research will help CR professionals to understand the utility of physical activity tracker devices such as Fitbit-Flex in their patients. A secondary aim is to estimate whether four days of data (combined two weekend days and two weekdays) or two days of data (two weekend days versus two weekdays) are needed to achieve acceptable reliability for Fitbit-Flex in measuring MVPA minutes and step counts.

## 2. Materials and Methods

### 2.1. Study Design

Data from a prospective repeated measures study were used to determine and compare the responsiveness of three different measures: exercise capacity (6MWT), functional activity (PASE) and free-living PA (Fitbit-Flex). This study is part of an ongoing funded trial called “Heart and Mind” designed to investigate the relationship between PA and cognitive function in CR patients. The data presented have not been reported previously.

### 2.2. Subjects and Setting

This study recruited a convenience sample from the CR programs at two tertiary hospital sites in Sydney, Australia. Typically, the CR programs within these hospitals involve six to eight weeks of supervised structured aerobic and resistance exercise for one hour, twice a week and a home-based program that aims for an accumulation of 30 min of exercise five days per week. The programs are tailored to the individual’s needs based on exercise capacity (6MWT) and an interview conducted by an exercise physiologist regarding existing exercise habits and barriers. 

Participants were eligible if they: (1) attended baseline assessment for CR; (2) were admitted for a diagnosis of CHD; (3) could participate in regular PA; (4) had no dementia diagnosis; (5) could understand English language sufficient to provide informed consent and undertake the questionnaires; (6) could participate in the full 16 weeks of the study, and (7) could wear and apply the Fitbit-Flex device. 

Forty-four participants consented to participate, with four withdrawing prior to commencing the trial due to re-hospitalization or leaving the region. Of the 40 participants who completed the CR entry assessment, 32 completed CR assessment and 26 completed CR follow-up assessment. Ninety percent of participants had completed Fitbit-Flex data collection for the required wear-time for the whole four-day study period at CR entry and 100% had completed data collection for the required wear-time at CR completion and CR follow-up assessments.

### 2.3. Data Collection and Measures

Assessments were performed at the entry of CR (baseline), the completion of CR (6–8 weeks), and at CR follow-up (16 weeks).

#### 2.3.1. Demographic and Clinical Data

Demographic and clinical details including anthropometric measures, medical history, and cardiovascular disease risk factors were collected from the participants and extracted from the CR program patient chart using a data collection form. Due to the small sample size in this study, these data were not used in analysis. 

#### 2.3.2. Physical Activity

The PA and exercise capacity of participants were assessed using three different measures:

- Fitbit-Flex; Participants’ PA levels were objectively measured using the Fitbit-Flex activity tracker (Fitbit Inc., San Francisco, CA, USA), a small and light accelerometer worn on the wrist. When initialized to the individual’s height and weight, Fitbit Flex uses a three-dimensional accelerometer to sense user movement to calculate steps walked and moderate–vigorous active minutes. Our validation study [15] previously demonstrated that Fitbit-Flex is a valid instrument for measuring daily step counts and MVPA minutes, and had high sensitivity, specificity, and positive predictive value for meeting PA guidelines in a CR population.
- The Physical Activity Scale for the Elderly; The self-reported PASE is a survey designed specifically to assess PA in older people [21]. Participants reported the frequency with which they participated over a one-week period in moderate and strenuous leisure activities including outdoor walking by indicating never, 1–2 days/week (seldom), 3–4 days/week (sometimes), or 5–7 days/week (often). Activity duration was indicated as either less than 1 h, between 1–2 h, 2–4 h, or more than 4 h. The final PASE minutes of MVPA were determined by multiplying the amount of time spent in each activity by item weight. The PASE was administered by a CR physiotherapist and has reported acceptable validity [22] and reliability [23], and was suitable for CR participants [12].
- Six-Minute Walk Test; Submaximal functional capacity was evaluated through the conventional 6MWT based on standards established by the American Thoracic Society [24]. The test was conducted by a physiotherapist. Before commencing the test, the participants were informed of the aims and how to rate their breathing and fatigue using the Borg Rating of Perceived Exertion Scale [25]. Participants were then instructed to commence walking around two cones 15 m apart as many times as possible and to inform the physiotherapist of any signs of discomfort. During the walk, the participants were given standardized encouragement. The 6MWT was measured twice with an intervening rest period of approximately 15 min, and the mean score was used in the analysis. The reliability and validity of the 6MWT in CR settings were previously established [26].

### 2.4. Procedure

Patients scheduled to begin CR were invited to participate in this study and informed of the study requirements. After eligibility was confirmed, written informed consent was obtained and the participants completed a self-administered questionnaire to collect socio-demographic information. Data on weight and height were extracted from the CR chart to initialize the Fitbit-Flex device. 

After initializing the device, each participant was provided with a brief written guide on device placement, wearing time, and how to troubleshoot any problems with the device. Participants were instructed to place the Fitbit-Flex on the non-dominant hand and to wear the device for four consecutive days (two weekend days and two weekdays) during waking hours (at least 10 h) except when showering or swimming. Participants were informed that data collection would be repeated at assessments at 8 and 16 weeks. All assessments took place in the same location as the baseline assessment. All participants were blinded to their Fitbit-Flex data throughout the study.

### 2.5. Data Analysis

Data analyses were performed using the Statistical Package for the Social Sciences, version 22. Mean scores, standard deviations, frequencies, and percentages were used to describe the socio-demographic and clinical characteristics of the sample. Both the relative change scores (i.e., (post − pre) ÷ pre-value) and the absolute change scores (i.e., paired *t*-tests) among the four measurements (i.e., Fitbit-Flex steps, Fitbit-Flex MVPA minutes, 6MWT meters, and PASE MVPA minutes) were used to compare change scores of the measures over time. Data were normally distributed, and parametric tests used. If any measures indicated statistically significant changes (based on paired *t*-test results, *p* < 0.05), then similarity in the responsiveness of the measure was compared using two additional statistical methods. First, comparing the magnitude of change for each measure with the expected magnitude of change due to participating in the CR program to ensure statistical significance was sufficient for clinical significance. Second, comparing the relative change (i.e., inspecting confidence interval (CI) overlap) to assess whether the measures were similarly responsive or if they measured different PA dimensions. When the units of the measures were similar (e.g., MVPA minutes as estimated by Fitbit-Flex and PASE), data can be examined by comparing their 95% CI. However, to our knowledge, there is currently no guideline available in the literature to examine differences in the responsiveness (no statistical significance) for two measures with different units (e.g., Fitbit-Flex steps and 6MWT meters). Thus, we inspected the 95% CIs of (the unitless) relative change for Fitbit-Flex steps and 6MWT meters to compare their change scores. Finally, intra-class correlation coefficients (ICCs) were used to estimate whether four or two days of data collection are needed to achieve acceptable reliability for Fitbit-Flex.

## 3. Results 

### 3.1. Sample Characteristics

The sample (N = 40) had a mean age of 66.2 ± 8.2 years and a mean body mass index of 27.9 ± 4.9 kg/m^2^. Most were males (67%), and 48% were employed. The sample was highly educated, with 63% of participants holding at least an undergraduate university degree. The most common cardiac diagnoses were myocardial infarction (70%), coronary artery disease (13%), and percutaneous coronary intervention (8%). Arthritis (28%) and asthma (18%) were among the common co-morbidities potentially impacting exercise participation.

### 3.2. Changes in the Measures over Time (Responsiveness)

All the measures demonstrated positive changes in PA following the CR intervention (Table 1). However, the relative change and the absolute change within each comparison period (i.e., entry of CR to completion of CR and entry of CR to CR follow-up) demonstrated that only measures of Fitbit-Flex steps and the 6MWT meters detected a significant initial response to the intervention. From the entry to the completion of CR, Fitbit-Flex detected a statistically significant increase in average daily steps (8024 to 9818; *p* = 0.015) and the 6MWT detected a statistically significant increase in the mean distance (477 to 516 m; *p* < 0.001). From the entry of CR to CR follow-up, only the 6MWT detected a sustained change (an increase in the 6MWT meters from 477 to 509 m; *p* < 0.001). In contrast, the measure of MVPA minutes estimated by Fitbit-Flex and PASE did not detect statistically significant changes from the entry of CR to completion of CR or from the entry of CR to CR follow-up.

### 3.3. Similarity in the Responsiveness of the Measures

**Figure 1:**
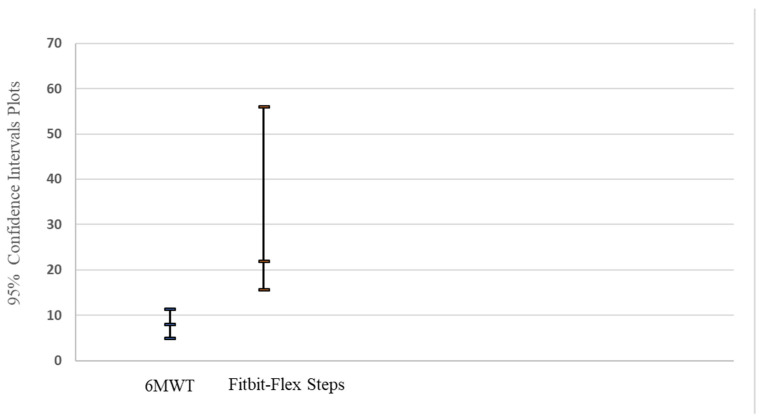
A 95% CI plot of the relative difference for 6MWT and Fitbit-Flex at entry of CR to completion of CR.

Only Fitbit-Flex steps and the 6MWT meters had similar change score patterns as they showed statistically significant increases following the CR program and significant relative change score improvements. Furthermore, at the entry of CR to completion of CR, the 95% CI of the relative change for Fitbit-Flex steps and the 6MWT meters did not overlap, suggesting different degrees of responsiveness (Figure 1).

### 3.4. Reliability of Fitbit-Flex in Estimating Step Counts and MVPA

When using two days of data (two weekdays vs. two weekend days) at the entry of CR and at the completion of CR, the ICCs for the reliability of Fitbit-Flex (Table 2) were moderately good in measuring step counts (ICC = 0.64 and 0.67, respectively) and MVPA minutes (ICC = 0.68 and 0.64, respectively), but at CR follow-up, two days of data were highly acceptable for measuring step counts and MVPA minutes (ICC = 0.85 and 0.74, respectively). However, the ICC values of Fitbit-Flex improved when four days of data (two weekdays and two weekend days) were used for measuring step counts at the entry of CR, completion of CR, and CR follow-up (ICC = 0.74, 0.79 and 0.90, respectively) and MVPA minutes (ICC = 0.75, 0.74 and 0.69, respectively).

## 4. Discussion

To our knowledge, this is the first study to compare the intervention responsiveness of four widely used PA measurements (i.e., Fitbit-Flex steps, Fitbit-Flex MVPA minutes, 6MWT meters, and PASE MVPA minutes) among CR participants over three different time periods. Results indicated that, unlike Fitbit-Flex, MVPA minutes and PASE MVPA minutes, the 6MWT meters and Fitbit-Flex steps showed significant and similar patterns of responsiveness to expected changes in PA due to participating in a CR program. Results also implied that the use of activity trackers in cardiac patients is a promising measurement tool for cardiac telerehabilitation. When wearing Fitbit-Flex, four days of measurements including weekend and weekday use, rather than two days only, were required to best determine CR participants’ typical PA levels. Notably, PA improved rapidly during CR program participation but declined relatively quickly once CR was complete.

Our findings revealed similar responsiveness between measures of the 6MWT meters and Fitbit-Flex steps from the entry of CR to completion of CR and from the entry of CR to CR follow-up. The increases observed in this study were in line with the 2% to 8% improvement reported by a systematic review [26] for repeated 6MWT (i.e., entry and completion of CR ranged from 301 to 489 m and from 377 to 555 m, respectively) in outpatient CR. Earlier studies reported improvements in the 6MWT meters at the completion of CR ranged from 40 to 74 m [27,28,29] which are also consistent in magnitude with the change found in our study (i.e., 40 m and 42 m improvement, respectively, at each assessment point). Likewise, in the present study, relative changes in the average Fitbit-Flex steps between assessment points were similar to those previously reported for CR participants. Our data showed the relative percent increase in step counts at each assessment point (22% and 4%) is comparable to an earlier CR study [30] (n = 64) that revealed the percentage increase in Fitbit-One steps was 22% from the entry of CR to one month and 19% from the entry of CR to three months. In addition, prior studies reported an average number of steps per day for cardiac patients between: (1) 7387 [31,32] and 8609 steps [33] at the entry of CR; (2) 6362 [30] and 9252.5 steps [34] at the completion of CR; and (3) 6186 [30] 7972 steps [35] at CR follow-up. Similarly, earlier studies reported average step counts increased by 1171 [30] and 2654 [36] from the entry of CR to the completion of CR, and by 497 [35] and 995 [30] from the entry of CR to CR follow-up. These results are consistent with the changes found in our study. These findings demonstrate that both measures showed responsiveness to the CR program. Importantly, the findings also indicate that PA is composed of multiple components and that both measures contribute important but different aspects to our understanding of changes in PA over time for CR patients. Hence, changes in functional capacity over time are captured by the 6MWT meters, whereas changes in PA levels over time are more appropriately evaluated by Fitbit-Flex steps.

Fitbit-Flex steps and the 6MWT meters both measured different PA dimensions. Fitbit-Flex contributes new and clinically relevant information related to the patient’s objectively measured PA changes over time, extending information provided on functional capacity by the conventional in-person 6MWT [37,38]. Fitbit-Flex provides tracking data for PA occurring in both the CR program setting and the free-living environment, acting as a continuously self-monitored progress indicator for both patients and clinicians [37,38]. Therefore, the sensor-based telemonitoring/tracking device overcomes the limitations associated with conventional measures which are typically applied at the beginning and the end of the rehabilitation program. Such measures fail to account for the changes in PA for the patient between the CR sessions, at their home, or their adherence to the CR guidelines. Considering that several CR programs are delivered through telehealth, self-assessed measures of PA and exercise capacity are needed. Hence, the Fitbit-Flex offers potential for telemonitored exercise-based CR.

Notably, PASE MVPA minutes and Fitbit-Flex MVPA minutes did not show significant responsiveness at the completion of CR nor during CR follow-up. The minutes of MVPA as measured by both Fitbit-Flex and PASE were higher than those reported for cardiac patients in comparable studies. Previous CR studies showed that minutes of MVPA increased from 15 to 25 [36] from the entry of CR to completion of CR (measured with a pedometer), between 25 [14] and 32 [32,39] at the completion of CR (measured with an accelerometer) and between 30 [40] and 38 [41] at the completion of CR (measured with self-reported questionnaires). Although most previous studies did not provide information about PA intensities and volume, the general consensus is that the majority of patients after completing a CR program failed to reach the recommended level of weekly MPVA minutes [39,42,43]. Our data showed the relative change of Fitbit-Flex MVPA minutes increased by 24% from the entry of CR to completion of CR and declined by 9% from CR entry to CR follow-up. These relative changes were also similar to the relative change recorded by Fitbit-One in an intervention study for postmenopausal women who had increased their minutes of MVPA by 11% from baseline to 4 weeks but had decreased their minutes by 14% at 16 weeks [44]. Nevertheless, it is not surprising that the estimation of Fitbit-Flex MVPA minutes was not consistent with the Fitbit-Flex total steps reported because the operationalized algorithms are different [45]. The manufacturer of Fitbit recently changed its tracker algorithm so that time spent in MVPA will be only counted if the wearer accumulates bouts of at least 10 min duration [45]. Hence, clinicians should be mindful that Fitbit-Flex steps are different from Fitbit-Flex MVPA minutes.

A close reading of the responsiveness of minutes of MVPA for both PASE and Fitbit-Flex at the completion of CR showed that the self-reported PASE response was approximately twice that of the Fitbit-Flex response. To our knowledge, there is no study reporting PASE minutes of MVPA for CR participants. However, our finding is similar to previous interventional studies which highlighted the measurement characteristics of PASE, and which reported an 18% increase in PASE between 3 and 6 months [46], and a 34% increase during two occasions separated by seven days [47]. Importantly, evidence shows PA levels in CR patients are much higher as measured by self-reported questionnaires than when measured objectively by monitoring devices, therefore limiting the usefulness of such questionnaires as a tool to assess progress [13]. The findings therefore suggest that clinicians should be aware of the limitations of the PASE questionnaire to differentiate between measurement error and real changes, thus diminishing the suitability of the questionnaire for measuring patients’ PA levels over time.

Variability in responsiveness was common in all evaluated measures. However, it is noteworthy that heterogeneity will vary depending on the population studied. Therefore, CR patients are not homogenous in regards to change, and it is likely that they change in a different manner [13]. Variations among participants occur in relation to age, sex, body size, disease severity, and stage of recovery. Hence, instead of excluding participants affected by these factors, we consider these factors to be representative of issues facing investigators working with the CR population [12,13]. Demonstrating that the Fitbit-Flex steps measure had different responsiveness compared to the 6MWT is important to highlight that both measures respond in different situations (i.e., a standardized environment for the 6MWT and a free-living environment for Fitbit-Flex). It is also important to demonstrate the purpose of the assessment because a measure that is suitable for one purpose may not necessarily be suitable for another. Nonetheless, future research is needed to understand the benefits of different types of PA following CR participation and to further explore methods for comparing the responsiveness of measures that have different units.

Our results estimated four monitoring days (i.e., combined of two weekdays and two weekend days) using Fitbit-Flex were required to best determine daily PA levels (i.e., step counts and minutes of MVPA) for CR participants at the entry of CR, completion of CR, and CR follow-up. The results of previous CR studies do not directly allow comparison with the current results because the same measure (i.e., Fitbit-Flex) was not used. However, Hertzog et al. [17] used a similar measure (i.e., RT3 accelerometer), but with non-comparable units of measurements (i.e., total energy expenditure, activity calories, and activity counts) for CR participants at 3 weeks, 6 weeks, and 3 months post-CR, and recommended three days (i.e., two weekdays and one weekend day) of data collection were required to monitor daily PA levels.

An important issue for CR program coordinators is that changes in PA achieved during CR may depend on participation and may not be sustained from one day to another. Measures of PA indicated that it improved during program participation but rapidly declined when participation ended except for the exercise capacity measure which indicated sustained change. Consistent with our analyses, PA levels—at least in terms of step counts—significantly increase on days patients attend CR compared to non-CR days [14,31,36,43]. In addition, PA levels achieved during CR decrease after completion of the program, with evidence showing only 28% to 30% of those who complete a CR program are still physically active after three months to one year [48,49,50]. We speculate that a lack of referral to the Phase-III CR program may relate to the rapid decline in PA levels post CR program. Nonetheless, to sustain long-term cardiovascular risk reduction benefits, efforts are required in maintaining PA levels in post-CR populations. Importantly, to encourage sustained PA, clinicians should promote PA outside the structured exercise session in the CR program, which is generally offered only three times per week [13]. A simple way to do this is to promote PA tracking.

We acknowledge that our study had a small sample size and that the participant attrition rate increased across the last two assessment points. The recruitment of a selected sample might also limit the generalizability of the results to other CHD populations. Moreover, a limitation in interpreting the results is that it is increasingly understood that PA increases during CR and decreases again afterward, so the lack of significant change may not be due entirely to the poor responsiveness of the measure. Conversely, the strength of this study was the inclusion of three time-point assessments to accurately compare the responsiveness of Fitbit-Flex, PASE and the 6MWT for CR participants during recovery. Another strength of this study was the inclusion of easily accessible innovative technology (Fitbit-Flex) which is valid, inexpensive, and easy for CR patients to use. Compliance with wearing Fitbit-Flex on CR days compared to non-CR days and variation in wear-time beyond the 10-h/day requested were not taken into account in the analyses and could lead to inconsistent comparisons between participants. Future research should address this issue.

## 5. Conclusions

Irrespective of whether PA assessment measures are performed conventionally in-person via a 6MWT, by self-report questionnaire, or using an activity tracker, it is important to understand the interrelationships among these measures and their similarities or differences in detecting changes in PA. Moreover, it is important to understand whether these measures appropriately support different models of CR delivery, including telehealth and virtual rehabilitation. The collective information provided by Fitbit-Flex steps and the 6MWT meters better reflect patients’ overall exercise capacity and PA than individual measures alone. The 6MWT meters are most accurate at evaluating changes in functional capacity at the completion of CR and CR follow-up, whereas Fitbit-Flex steps are the best at capturing free-living PA and differentiating it from functional capacity at the completion of CR. This provides an additional measure, step counts, of use to assess outcomes following cardiac rehabilitation programs. The Fitbit-Flex activity tracker and the self-reported PASE estimates of MVPA minutes have limited capacity to differentiate between measurement error and real change. Therefore, we recommend that Fitbit-Flex step counts be used in addition to the conventional 6MWT to provide a more comprehensive understanding of patients’ activities occurring outside of CR. Moreover, data from the present study provide further support of the benefits of Fitbit-Flex steps as a promising tool to track PA levels both in CR settings and for cardiac telerehabilitation programs. This is because it provides continuous PA data which assist clinicians to monitor patients’ adherence to CR guidelines, in personalizing treatment plans, and in initiating timely interventions when needed. For the best estimates of the usual pattern of PA, the use of Fitbit-Flex should include two weekend days and two weekdays.

## Figures and Tables

**Table 1:** Responsiveness of Fitbit-Flex, 6MWT and PASE at entry of CR, completion of CR, and CR follow-up.

Characteristic	Entry of CR (N = 32) Mean (SD)	Completion of CR (N = 32) Mean (SD)	Absolute Difference	Relative Difference (95% CI)	*p*-Value	Entry of CR (N = 26) Mean (SD)	CR Follow-Up (N = 26) Mean (SD)	Absolute Difference	Relative Difference (95% CI)	*p*-Value
6-min walk test (meters)	476.50 (85.06)	515.96 (95.71)	39.46	8% (5.06, 11.74)	0.001	467.27 (72.47)	508.81 (79.46)	41.54	9% (5.75, 12.47)	0.001
Fitbit-Flex MVPA min/d (average 4 days)	33.33 (20.51)	41.42 (28.11)	8.09	24% (−7.32, 71.29)	0.142	32.89 (21.62)	30.02 (27.53)	−2.87	9% (−39.42, 29.42)	0.581
PASE MVPA min/d (average 7 days)	48.97 (54.86)	64.10 (69.33)	15.12	31% (−13.16, 40.12)	0.153	51.40 (56.09)	57.24 (57.41)	5.84	12% (−15.51, 48.47)	0.549
Fitbit-Flex steps/d (average 4 days)	8024.44 (2642.25)	9818.20 (3257.57)	1793.76	22% (15.65, 55.99)	0.015	8032.99 (2839.03)	8361.49 (3686.67)	328.50	4% (−8.47, 26.18)	0.562

Relative change is (post–pre)/pre-value; PASE: Physical Activity Scale for the Elderly; 6MWT: Six-Minute Walk Test; CR: cardiac rehabilitation; MVPA: moderate–vigorous physical activity.

**Table 2:** Estimated ICCs for Fitbit-Flex for measuring step counts and minutes of MVPA.

Length of Data Collection						
Characteristic	Entry of CR		Completion of CR		CR Follow-Up	
	2 Weekdays vs. 2 Weekend Days	Combined Weekdays and Weekend Days	2 Weekdays vs. 2 Weekend Days	Combined Weekdays and Weekend Days	2 Weekdays vs. 2 Weekend Days	Combined Weekdays and Weekend Days
MVPA minutes	0.68 (0.39, 0.85)	0.75 (0.58, 0.86)	0.64 (0.25, 0.82)	0.74 (0.55, 0.86)	0.74 (0.43, 0.88)	0.69 (0.44, 0.84)
Steps	0.64 (0.26, 0.82)	0.74 (0.57, 0.85)	0.67 (0.34, 0.84)	0.79 (0.64, 0.89)	0.85 (0.67, 0.93)	0.90 (0.82, 0.95)

ICCs: intra-class correlation coefficients; CR: cardiac rehabilitation; MVPA: moderate–vigorous physical activity.

## Data Availability

The data presented in this study are available in this article.
